# The 2024 Nobel Prize in Physiology or Medicine: microRNA Takes Center Stage

**DOI:** 10.3390/ncrna10060062

**Published:** 2024-12-12

**Authors:** George A. Calin, Florent Hubé, Michael R. Ladomery, Nicholas Delihas, Manuela Ferracin, Laura Poliseno, Luca Agnelli, Suresh K. Alahari, Ai-Ming Yu, Xiao-Bo Zhong

**Affiliations:** 1 Department of Translational Molecular Pathology, Center for RNA Interference and Non-Coding RNAs, University of Texas MD Anderson Cancer Center, Houston, TX 77030, USA; 2 Laboratoire Biologie du Développement, Institut de Biologie Paris-Seine, Transgenerational Epigenetics & Small RNA Biology, Sorbonne Université, CNRS, UMR7622, 75005 Paris, France; 3 School of Applied Sciences, University of the West of England, Bristol BS16 1QY, UK; 4 Department of Microbiology and Immunology, Renaissance School of Medicine, Stony Brook University, Stony Brook, NY 11794, USA; 5 Department of Medical and Surgical Sciences (DIMEC), University of Bologna, via S. Giacomo, 14, 40126 Bologna, Italy; 6 National Research Council (CNR) and Oncogenomics Unit, Core Research Laboratory (CRL), Institute of Clinical Physiology (IFC), Istituto per lo Studio, la Prevenzione e la Rete Oncologica (ISPRO), via Moruzzi 1, 56124 Pisa, Italy; 7 Department of Diagnostic Innovation, Fondazione IRCCS Istituto Nazionale dei Tumori, Via Venezian 1, 20133 Milan, Italy; 8 Medical Oncology, Fondazione IRCCS Istituto Nazionale dei Tumori, Via Venezian 1, 20133 Milan, Italy; 9 Department of Biochemistry and Molecular Biology, LSU School of Medicine, New Orleans, LA 70112, USA; 10 Department of Biochemistry and Molecular Medicine, School of Medicine, University of California at Davis, Sacramento, CA 95817, USA; 11 Department of Pharmaceutical Sciences, School of Pharmacy, University of Connecticut, Storrs, CT 06269, USA

## 1. George A. Calin—Professor, M.D. Anderson Cancer Center

The *Non-coding* Journal Editorial Board Members would like to congratulate Victor Ambros and Gary Ruvkun, who were jointly awarded the 2024 Nobel Prize in Physiology or Medicine for their groundbreaking discovery of microRNAs and the role of microRNAs in post-transcriptional gene regulation, uncovering a previously unknown layer of gene control in eukaryotes.

Their discovery of miRNAs and the elucidation of their role in gene regulation had a huge impact on the field of molecular biology and our understanding of various biological processes. With the announcement of Nobel Prize winners comes a time for the celebration of outstanding science, as well as a time to contemplate the further translational potential such molecules. Several Editorial Board Members wished to express their thoughts about the significance of this fundamental discovery, which has changed the way we think about biology and medicine.

Let us start first with a brief summary of the two laureates and their work. 

Victor Ambros graduated from MIT in 1975. While at MIT, he conducted poliovirus research under Nobel laureate David Baltimore. He continued his research as a postdoctoral researcher in the Nobel laureate H. Robert Horvitz’s lab at MIT, where he shifted his research focus towards genetic pathways in the nematode *C. elegans*, specifically focusing on the regulation of developmental timing. He continued his research during his tenures at Harvard and Dartmouth, and he now works at the University of Massachusetts Medical School. In 1993, Ambros and his team discovered that miRNAs work as small non-coding regulatory RNAs through their studies on the *lin-4* gene in *C. elegans*. They found that lin-4 controls the protein *LIN-14* via a small RNA mechanism, marking the first identification of a miRNA (lin-4), a class of molecules now known to regulate gene expression post-transcriptionally across species [1]. For his seminal contributions, Ambros was accepted into the National Academy of Sciences in 2007 and to the American Academy of Arts and Sciences in 2011.

Gary Ruvkun earned his BA in biophysics from UC Berkeley in 1973, before going on to earn a PhD in biophysics from Harvard in 1982. He conducted postdoctoral research with two Nobelists, Robert Horvitz at MIT and Walter Gilbert at Harvard. His group’s work—coinciding with the work of Ambros’s group, to the point where his group’s work and that of Ambros’s group were published in the same Cell issue—revealed that lin-4 miRNA regulates the translation of target messenger RNAs by binding imperfectly to complementary sequences [2]. Ruvkun continued to make groundbreaking discoveries on microRNAs by finding that let-7, the second cloned miRNA, is well conserved across diverse species, unveiling RNA-based regulation that extends well beyond *C. elegans* [3]. Ruvkun’s research also focuses on understanding the insulin signaling pathways in C. elegans and their role in metabolism and aging [4]. For his seminal contributions, Ruvkun was elected to the National Academy of Sciences in 2008.

## 2. Florent Hubé—Staff Scientist, Sorbonne Université, CNRS

In the 1990s, while Ambros and Ruvkun were independently studying *Caenorhabditis elegans*, they made a groundbreaking discovery. Ambros investigated the *lin-4* gene, while Ruvkun focused on *lin-14*. The intriguing part is that *lin-4* regulates *lin-14* in an unusual way, but the mechanism remained a mystery for years.

In 1993, Ambros discovered that *lin-4* did not code for a protein, but for a small non-coding RNA, now known as a microRNA (miRNA). This miRNA bound to the *lin-14* messenger RNA (mRNA), regulating its translation. Simultaneously, Ruvkun demonstrated that *lin-4* interacted with specific sequences in the 3′ untranslated region of the *lin-14* mRNA, inhibiting protein production.

The question then arose whether or not this mechanism was unique to *C. elegans* or if similar miRNAs existed in other organisms. During an informal discussion, Rosalind Lee proposed the bold idea that miRNAs might also exist in more complex organisms, including humans. Although this seemed improbable at the time, Ambros encouraged Lee to pursue the hypothesis. They eventually found that miRNAs were evolutionarily conserved and played a crucial role in gene regulation across many species, including mammals [1].

Ambros’s discovery, alongside previous findings regarding *E. Coli* from the labs of Inouye and Delihas, revolutionized molecular biology by revealing that RNA could regulate genes. The first evidence came from *E. coli*, where small RNAs like micF were shown to control gene expression, challenging the idea that only proteins and mRNAs played regulatory roles [5]. The identification of miRNAs unveiled a new class of non-coding RNAs essential for development and physiology.

What makes this discovery even more remarkable is that Ambros and Ruvkun, though working on different aspects, reached complementary conclusions simultaneously. This highlights how independent research can converge to produce revolutionary insights. 

## 3. Michael R. Ladomery—Professor, University of the West of England

The 2024 Nobel Prize in Physiology or Medicine was deservedly awarded to two brilliant RNA biologists, Victor Ambros and Gary Ruvkun, for their discovery of miRNAs and their pivotal role in controlling gene expression. I was introduced to the wonderful world of molecular biology as an undergraduate in the late 1980s, and at this time, relatively little was known about post-transcriptional control of gene expression. We knew of the existence of various post-transcriptional processes, including alternative splicing (though we were not aware of its widespread importance), as well as mRNA translation control and mRNA stability and localization, being aware that they are all regulated processes. However, it never occurred to us, in those days, that there could be actual eukaryotic RNA molecules that themselves function as direct regulators of gene expression. Ambros and Ruvkun studied the genetic control of developmental timing in the nematode *C.elegans*. Their work clearly showed that the product of the *lin-4* gene in the nematode *C.elegans* negatively regulates the *lin-14* gene, with the latter encoding a protein. To their surprise, however, *lin-4* clearly did not express a protein, but rather a small RNA molecule (a miRNA). But this went against the grain of what was known about gene regulation in eukaryotes. Here, they displayed the brilliance and determination that drive significant scientific advances and persevered in their experiments, working out the underlying mechanism. They showed that the lin-4 mRNA bound directly to target sequences in the *lin-14* mRNA, repressing its translation. Their experiments combined brilliant genetics and biochemistry and resulted in two seminal *Cell* papers in 1993. At first, there was a degree of skepticism, or perhaps a sense that the existence of eukaryotic small regulatory RNAs was a bit of a quirk. We now know that it was far from a quirk and that there are multiple miRNAs that help fine-tune gene expression in development and in disease. MiRNAs are widespread and vitally important across both animal and plant kingdoms. The lesson for that young scientists can learn from Ambros and Ruvkun’s brilliant work is clear. Follow the evidence! Be creative, persevere, and be open to new ideas. After all, nature has had hundreds of millions of years to experiment and come up with new mechanisms to regulate gene expression. 

## 4. Nicholas Delihas—Professor Emeritus, Stony Brook University

This year, the Nobel Committee highlighted the discovery of miRNAs and their role in post-transcriptional gene regulation in the nematode *C. elegans*, work published in 1993, over 30 years ago. Since the emergence of this research, we have learned of the enormous significance of miRNAs and their functions in animals’ molecular/cell processes, including disease, so recognition is indeed due. But it is important to consider miRNAs within the context of the history of regulation by RNAs. This shows a richer and broader picture of regulation. The first experimental demonstration of a regulatory RNA gene and its transcript regulating the expression of another gene via the process of regulatory RNA/target mRNA base pairing came in the form of studies using *E*. *coli* bacteria, reported five years before the *C. elegans*-based miRNA work [5,6,7]. This *E. coli*-based work opened the door to reveal a fundamental principle in molecular biology: RNA can regulate gene expression. Together with the miRNA studies and with additional findings regarding regulatory RNA genes in archaebacteria, protists, fungi, and viruses [8,9], the universal principle in molecular biology emerged: RNAs regulate gene expression in all life forms, prokaryotes, eukaryotes, and viruses. Adding to this, there are the important tRNA fragments that also function in regulating gene expression [10,11]. Including lncRNAs and circular RNAs as regulatory molecules involved in numerous molecular processes, this represents an astounding picture of broad regulation by ncRNAs. Nevertheless, first and foremost, one must honor the groundbreaking work on RNA-based regulation by Jun-ichi Tomizawa, who demonstrated the first example of regulation via regulatory RNA/target RNA base pairing with the control of ColEl DNA Replication by RNA [12]. Tomizawa’s findings may be looked at as representing the root of the “historical tree of regulatory ncRNAs”. Thus, there has been a remarkable advance, from the 1950s, 1960s, when RNA was essentially considered functionless, but this concept of RNA started to change with the much-heralded discovery of messenger RNA [13].

## 5. Manuela Ferracin—Professor, University of Bologna

The discovery that miRNAs act as fundamental post-transcriptional regulators [1,2] revolutionized our understanding of protein expression regulation and provided answers to many unresolved questions in cell biology. It was like peeling back a layer and uncovering a new level of complexity within cells, deeply interconnected with other molecular players. 

The discovery of miRNAs and their activity transformed our knowledge of the molecular mechanisms underlying the pathogenesis of human diseases. From the 2000s onwards, many research groups worldwide began investigating the differences in miRNA expression between diseased and healthy states, finding that miRNA dysregulation is a feature of all diseased cells. Later, researchers studied the complex regulatory networks involving miRNAs, target genes, and other long non-coding RNAs, providing a picture that became clearer every day [14].

MiRNAs have been widely investigated as disease biomarkers, and their manipulation in vitro and in vivo has proven to be both feasible and powerful. However, there is still a long way to go before every aspect of miRNAs’ activity and role is clarified and their potential is fully exploited. And we will not just stand by and watch. We will be at the forefront. 

## 6. Laura Poliseno—Senior Staff Scientist, Institute of Clinical Physiology, CNR

This is exactly what biological sciences are for, attributing a functional meaning to new classes of molecules and uncovering new layers to the regulation of gene expression. The awarding of the Nobel Prize to Prof. Ambros and Prof. Ruvkun is first and foremost a recognition of their ability to think out of the box and to look for biological functions outside an aminoacidic sequence. It also contributes to freeing non-coding RNAs from the unspoken perception of an “inferiority complex” relative to proteins. Finally, it is an incentive for all of us to persevere in our research activities. We must all continue to uncover the hidden secrets of our favorite class of non-coding RNAs with renewed enthusiasm. 

## 7. Luca Agnelli—Senior Researcher, IRCCS National Cancer Institute

Among bioinformatics researchers, the question that circulated a few years ago was not ‘whether’ the discoverers of miRNAs would receive the Nobel Prize, but ‘when’ they would. Since their discovery, high-throughput technologies and continually evolving analysis pipelines have been a crucial source of miRNA data for the scientific community. Following the initial detection systems based on Q-RT-PCR and microarray technologies, the advent of Next-Generation Sequencing (NGS) has led to an exponential increase in miRNA data volume, presenting both challenges and opportunities for bioinformatics.

Advances in bioinformatics have been essential for managing, analyzing, and interpreting these vast data, as skillfully described in a recent review by Buitrago et al. [15], published in the *ncRNA* journal. The typical bioinformatic analysis of miRNA sequencing data involves not only conventional steps such as data preprocessing, mapping, and annotation, but also critical tasks for functional miRNA interpretation. Differential expression analysis, for example, must be paired with sequence feature analysis, novel miRNA prediction, and the analysis of sequence variation in mature miRNA isoforms [16]. Furthermore, downstream analyses such as target prediction and the study of miRNAs in the context of transcriptional regulatory networks, pathways, and diseases are equally indispensable (e.g., miR2Disease, OncomiR [17,18]).

One of the bioinformatic cornerstone techniques developed for miRNA research is in silico miRNA target prediction. This approach uses computational algorithms to predict potential mRNA targets based on sequence complementarity and thermodynamic stability, providing valuable insights into the regulatory roles of miRNAs. Among the most used tools for this purpose are TargetScan [19], RNA22 [20], and the TarBase family.

Further complexities in miRNA functions were uncovered with the identification of unconventional miRNA isoforms, known as isomiRs. These were first documented in 2008, identified through robust analysis of high-throughput sequencing data [21]. IsomiRs, which arise from variations in miRNA processing or modifications, expand the diversity of miRNA molecules within a cell, suggesting a more refined modulation of gene expression. Bioinformatics tools such as IsoMiRmap [22], isomiRID [23], and miR&moRe2 [24], specifically designed to identify and catalog these isomiRs, have become indispensable in understanding their role and prevalence across various biological contexts.

Moreover, bioinformatics approaches have significantly enhanced the study of miRNA regulatory networks. By integrating miRNA, mRNA, and other non-coding RNA expression profiles, researchers can reconstruct intricate networks that illustrate the dynamic regulatory relationships mediated by miRNAs. One of the most promising discoveries supported by bioinformatics and later validated experimentally was the competing endogenous RNA (ceRNA) relationship between miRNAs and non-coding RNA species [25]. These analyses not only shed light on the biological processes and pathways regulated by miRNAs but also help identify key nodal miRNAs that could serve as therapeutic targets or biomarkers, with relevance for human diseases and cancer [26].

However, the exponential growth in miRNA-related publications and interest warrants caution and imposes several categorical imperatives: accuracy, scientific rigor in data analysis and interpretation, and, importantly, the need for regularly updated procedures [27]. The vast majority of miRNA data have been derived from in silico observations, and miRNA research has been heavily shaped by bioinformatics, from managing large datasets to uncovering the finer details of miRNA regulation. This reliance on computational methods carries the risk of generating significant background noise and false-positive results. Therefore, increased attention in the evaluation of research findings is necessary, along with functional validations aimed at studying basic mechanisms and paving the way for translational outcomes and, in human contexts, desirable clinical applications. 

## 8. Suresh K. Alahari—Professor, Louisiana State University Health Sciences Center

The advent of the miRNAs in the early 1990s has proven to be a tremendously significant development within the purview of gene regulation. They participate in the regulation of a broad assembly of processes vital to proper cell function and the perturbation of these pathways following alteration of miRNA expression is strongly believed to contribute to the pathogenesis of cancer. A large body of evidence indicates that miRNAs regulate the expression of different genes that play an important role in cancer cell invasion, migration, and metastasis. Given the importance of miRNAs in regulating cellular differentiation and proliferation, it is not surprising that their misregulation is linked to cancer. In cancer, miRNAs function as regulatory molecules, acting as oncogenes or tumor suppressors. Amplification or overexpression of miRNAs can down-regulate tumor suppressors or other genes involved in cell differentiation, thereby contributing to tumor formation by stimulating proliferation, angiogenesis, and invasion, i.e., they act as oncogenes. Similarly, miRNAs can down-regulate different proteins with oncogenic activity, i.e., they act as tumor suppressors. We published 11 papers on miRNAs and their importance in cancer, and it was only possible because of the discovery of miRNAs by Victor Ambros, Gary Avruskin, and others [1,2]. In our study, we demonstrated the importance of miRNA-27b, 23b, miR-149, let-7a, let-7d, and miR-532 [28,29,30,31,32,33,34,35,36,37,38]. We are currently examining the prognostic importance of miRNAs in cancer, and this research will further emphasize the importance of miRNA in clinical settings, which will further strengthen the 2024 Nobel Prize-winning research. We are confident that many more outstanding contributions to the field of miRNA will be forthcoming from various scientists across the globe. 

## 9. Ai-Ming Yu—Professor, University of California-Davis; Xiao-Bo Zhong—Professor, University of Connecticut

Interindividual differences are common in the biological processes of absorption, distribution, metabolism, and excretion (ADME) or the pharmacokinetics (PK) of drugs, and excessive variations may cause therapeutic failure or severe adverse events during clinic therapy. Genetic polymorphisms [39,40], epigenetic mechanisms [3,4,5,41,42,43], and xenobiotic receptor-controlled transcriptional regulation [44,45,46] of the genes encoding key proteins involved in the ADME/PK processes significantly contribute to excessive variations which could consequently alter drug efficacy or safety. In addition to those known mechanisms, post-transcriptional regulation of the ADME genes by miRNAs (miRNAs) has also shown a big impact on the variability of ADME/PK [47,48,49]. These findings should appreciate the fundamental discovery of functional miRNAs derived from the genome to govern post-transcriptional gene regulation by Dr. Victor Ambros and Dr. Gary Ruvkun [1,2], who have been awarded the Nobel Prize in Physiology or Medicine for 2024. Non-coding miRNAs have been found to be involved in the post-transcriptional regulation of genes in various ADME/PK pathways, including Phase I drug-metabolizing enzymes such as cytochrome P450s (as well as CYPs, including CYP1A2 [50], CYP1B1 [51], CYP2E1 [52], and CYP3A4 [53]); Phase II drug-metabolizing enzymes such as UGT1A1 [54,55] and SULT2A1 [56]; and uptake and efflux transporters such as ABCC1 [57], ABCG2 [58,59], and OATP1B1 [60]. For instance, miR-27b-3p has been revealed to control the protein outcomes of CYP1B1 [51] and CYP3A4 [53], as well as the critical gene regulatory factor vitamin D receptor (VDR or NR1I1) [61], through interacting with their 3′-untranslated regions (3′UTR), leading to remarkable changes in drug-metabolizing capacities. As another example, the breast cancer resistance protein (BCRP or ABCG2), one of the ATP-binding cassette transporters critical for cellular drug transport, is subject to post-transcriptional gene regulation by a number of miRNAs, including miR-328–3p [58] and miR-519c-3p [62], which can thus be utilized to effectively enhance intracellular drug accumulation and subsequently sensitize carcinoma cells to the drug [63]. Therefore, research on miRNA-controlled post-transcriptional gene regulation of clinically important ADME genes not only advances the understanding of novel mechanisms underlying variable ADME/PK processes but also offers clues for the development of new therapeutic strategies.

## References

[1] Lee R.C., Feinbaum R.L., Ambros V. (1993). The *C. elegans* heterochronic gene lin-4 encodes small RNAs with antisense complementarity to lin-14. Cell.

[2] Wightman B., Ha I., Ruvkun G. (1993). Posttranscriptional Regulation of the Heterochronic Gene Lin-14 by Lin-4 Mediates Temporal Pattern Formation in *C. elegans*. Cell.

[3] Pasquinelli A., Reinhart B., Slack F., Mailer B., Kuroda M., Martin-dale M., Srinivasan A., Fishman M., Hayward D., Ball E. (2000). Conservation across animal phylogeny of the sequence and temporal expression of the 21 nucleotide let-7 heterochronic regulatory RNA. Nature.

[4] Kimura K.D., Tissenbaum H.A., Liu Y., Ruvkun G. (1997). daf-2, an insulin receptor-like gene that regulates longevity and diapause in Caenorhabditis elegans. Science.

[5] Andersen J., Delihas N., Ikenaka K., Green P.J., Pines O., Ilercil O., Inouye M. (1987). The isolation and characterization of RNA coded by the micF gene in *Escherichia coli*. Nucleic Acids Res..

[6] Andersen J., Forst S.A., Zhao K., Inouye M., Delihas N. (1989). The function of micF RNA. micF RNA is a major factor in the thermal regulation of OmpF protein in *Escherichia coli*. J. Biol. Chem..

[7] Delihas N. (1995). Regulation of gene expression by trans-encoded antisense RNAs. Mol. Microbiol..

[8] Cross S.T., Michalski D., Miller M.R., Wilusz J. (2019). RNA regulatory processes in RNA virus biology. Wiley Interdiscip. Rev. RNA.

[9] Ruivinho C., Gama-Carvalho M. (2023). Small non-coding RNAs encoded by RNA viruses: Old controversies and new lessons from the COVID-19 pandemic. Front. Genet..

[10] Raina M., Ibba M. (2014). tRNAs as regulators of biological processes. Front. Genet..

[11] Magee R., Rigoutsos I. (2020). On the expanding roles of tRNA fragments in modulating cell behavior. Nucleic Acids Res..

[12] Tomizawa J. (1984). Control of cole 1 plasmid replication: The process of binding of RNA I to the primer transcript. Cell.

[13] Brenner S., Jacob F., Meselson M. (1961). An Unstable Intermediate Carrying Information from Genes to Ribosomes for Protein Synthesis. Nature.

[14] Nemeth K., Bayraktar R., Ferracin M., Calin G.A. (2024). Non-coding RNAs in disease: From mechanisms to therapeutics. Nat. Rev. Genet..

[15] Buitrago D.L., Lovering R.C., Caporali A. (2023). Insights into Online miRNA Bioinformatics Tools. Noncoding RNA.

[16] Bortolomeazzi M., Gaffo E., Bortoluzzi S. (2019). A survey of software tools for miRNA discovery and characterization using RNA-seq. Brief. Bioinform..

[17] Wong N.W., Chen Y., Chen S., Wang X. (2018). OncomiR: An online resource for exploring pan-cancer miRNA dysregulation. Bioinformatics.

[18] Jiang Q., Wang Y., Hao Y., Juan L., Teng M., Zhang X., Li M., Wang G., Liu Y. (2009). miR2Disease: A manually curated database for miRNA deregulation in human disease. Nucleic Acids Res..

[19] McGeary S.E., Lin K.S., Shi C.Y., Pham T.M., Bisaria M., Kelley G.M., Bartel D.P. (2019). The biochemical basis of miRNA targeting efficacy. Science.

[20] Miranda K.C., Huynh T., Tay Y., Ang Y.-S., Tam W.-L., Thomson A.M., Lim B., Rigoutsos I. (2006). A pattern-based method for the identification of MiRNA binding sites and their corresponding heteroduplexes. Cell.

[21] Morin R.D., O’Connor M.D., Griffith M., Kuchenbauer F., Delaney A., Prabhu A.-L., Zhao Y., McDonald H., Zeng T., Hirst M. (2008). Application of massively parallel sequencing to miRNA profiling and discovery in human embryonic stem cells. Genome Res..

[22] Loher P., Karathanasis N., Londin E., Bray P.F., Pliatsika V., Telonis A.G., Rigoutsos I. (2021). IsoMiRmap: Fast, deterministic and exhaustive mining of isomiRs from short RNA-seq datasets. Bioinformatics.

[23] Valter de Oliveira L.F., Christoff A.P., Margis R. (2013). isomiRID: A framework to identify miRNA isoforms. Bioinformatics.

[24] Gaffo E., Bortolomeazzi M., Bisognin A., Di Battista P., Lovisa F., Mussolin L., Bortoluzzi S. (2020). MiR&moRe2: A Bioinformatics Tool to Characterize miRNAs and miRNA-Offset RNAs from Small RNA-Seq Data. Int. J. Mol. Sci..

[25] Salmena L., Poliseno L., Tay Y., Kats L., Pandolfi P.P. (2011). A ceRNA hypothesis: The Rosetta Stone of a hidden RNA language?. Cell.

[26] Dragomir M., Mafra A.C.P., Dias S.M.G., Vasilescu C., Calin G.A. (2018). Using miRNA Networks to Understand Cancer. Int. J. Mol. Sci..

[27] Chen L., Heikkinen L., Wang C., Yang Y., Sun H., Wong G. (2019). Trends in the development of miRNA bioinformatics tools. Brief. Bioinform..

[28] Wang Y., Rathinam R., Walch A., Alahari S.K. (2009). ST14 (suppression of tumorigenicity 14) gene is a target for miR-27b, and the inhibitory effect of ST14 on cell growth is independent of miR-27b regulation. J. Biol. Chem..

[29] Baranwal S., Alahari S.K. (2010). miRNA control of tumor cell invasion and metastasis. Int. J. Cancer.

[30] Shenouda S.K., Alahari S.K. (2009). MicroRNA function in cancer: Oncogene or a tumor suppressor?. Cancer Metastasis Rev..

[31] Jain P., Alahari S.K. (2011). Breast cancer stem cells: A new challenge for breast cancer treatment. Front. Biosci..

[32] Jin L., Wessely O., Marcusson E.G., Ivan C., Calin G.A., Alahari S.K. (2013). Prooncogenic factors miR-23b and miR-27b are regulated by Her2/Neu, EGF, and TNF-α in breast cancer. Cancer Res..

[33] Eastlack S.C., Alahari S.K. (2015). MicroRNA and Breast Cancer: Understanding Pathogenesis, Improving Management. Noncoding RNA.

[34] O’Bryan S., Dong S., Mathis J.M., Alahari S.K. (2017). The roles of oncogenic miRNAs and their therapeutic importance in breast cancer. Eur. J. Cancer.

[35] Eastlack S.C., Dong S., Ivan C., Alahari S.K. (2018). Suppression of PDHX by microRNA-27b deregulates cell metabolism and promotes growth in breast cancer. Mol. Cancer.

[36] Yousefi H., Poursheikhani A., Bahmanpour Z., Vatanmakanian M., Taheri M., Mashouri L., Alahari S.K. (2020). SARS-CoV infection crosstalk with human host cell noncoding-RNA machinery: An in-silico approach. Biomed. Pharmacother..

[37] Drula R., Pardini B., Fu X., De Los Santos M.C., Jurj A., Pang L., El-Daly S.M., Fabris L., Knutsen E., Dragomir M.P. (2023). 17β-estradiol promotes extracellular vesicle release and selective miRNA loading in ERα-positive breast cancer. Proc. Natl. Acad. Sci. USA.

[38] Mahajan K., Das A.V., Alahari S.K., Pothuraju R., Nair S.A. (2024). MicroRNA-532-3p Modulates Colorectal Cancer Cell Proliferation and Invasion via Suppression of FOXM1. Cancers.

[39] Grossman I. (2009). ADME Pharmacogenetics: Current Practices and Future Outlook. Expert. Opin. Drug Metab. Toxicol..

[40] Deb S., Hopefl R., Reeves A.A., Cvetkovic D. (2024). ADME Gene-Related Pharmacogenomic Labeling of FDA-Approved Drugs: Comparison with Clinical Pharmacogenetics Implementation Consortium (CPIC) Evidence Levels. Medicines.

[41] Ingelman-Sundberg M., Zhong X.-B., Hankinson O., Beedanagari S., Yu A.-M., Peng L., Osawa Y. (2013). Potential Role of Epigenetic Mechanisms in the Regulation of Drug Metabolism and Transport. Drug Metab. Dispos..

[42] Yu A.-M., Ingelman-Sundberg M., Cherrington N.J., Aleksunes L.M., Zanger U.M., Xie W., Jeong H., Morgan E.T., Turnbaugh P.J., Klaassen C.D. (2017). Regulation of Drug Metabolism and Toxicity by Multiple Factors of Genetics, Epigenetics, LncRNAs, Gut Microbiota, and Diseases: A Meeting Report of the 21st International Symposium on Microsomes and Drug Oxidations (MDO). Acta Pharm. Sin. B.

[43] Jin J., Zhong X. (2023). Epigenetic Mechanisms Contribute to Intraindividual Variations of Drug Metabolism Mediated by Cytochrome P450 Enzymes. Drug Metab. Dispos..

[44] Tolson A.H., Wang H. (2010). Regulation of Drug-Metabolizing Enzymes by Xenobiotic Receptors: PXR and CAR. Adv. Drug Deliv. Rev..

[45] Mackowiak B., Wang H. (2016). Mechanisms of Xenobiotic Receptor Activation: Direct vs. Indirect. Biochim. Et. Biophys. Acta (BBA)—Gene Regul. Mech..

[46] Wallace B.D., Redinbo M.R. (2013). Xenobiotic-Sensing Nuclear Receptors Involved in Drug Metabolism: A Structural Perspective. Drug Metab. Rev..

[47] Nakano M., Nakajima M. (2018). Current Knowledge of MiRNA-Mediated Regulation of Drug Metabolism in Humans. Expert. Opin. Drug Metab. Toxicol..

[48] Yu A.-M., Tian Y., Tu M.-J., Ho P.Y., Jilek J.L. (2016). MiRNA Pharmacoepigenetics: Posttranscriptional Regulation Mechanisms behind Variable Drug Disposition and Strategy to Develop More Effective Therapy. Drug Metab. Dispos..

[49] Yu A.-M., Pan Y.-Z. (2012). Noncoding MiRNAs: Small RNAs Play a Big Role in Regulation of ADME?. Acta Pharm. Sin. B.

[50] Chen Y., Zeng L., Wang Y., Tolleson W.H., Knox B., Chen S., Ren Z., Guo L., Mei N., Qian F. (2017). The Expression, Induction and Pharmacological Activity of CYP1A2 Are Post-Transcriptionally Regulated by MiRNA Hsa-MiR-132–5p. Biochem. Pharm..

[51] Tsuchiya Y., Nakajima M., Takagi S., Taniya T., Yokoi T. (2006). MiRNA Regulates the Expression of Human Cytochrome P450 1B1. Cancer Res..

[52] Wang Y., Yu D., Tolleson W.H., Yu L.-R., Green B., Zeng L., Chen Y., Chen S., Ren Z., Guo L. (2017). A Systematic Evaluation of MiRNAs in Regulating Human Hepatic CYP2E1. Biochem. Pharm..

[53] Pan Y.-Z., Gao W., Yu A.-M. (2009). MiRNAs Regulate CYP3A4 Expression via Direct and Indirect Targeting. Drug Metab. Dispos..

[54] Dluzen D.F., Sun D., Salzberg A.C., Jones N., Bushey R.T., Robertson G.P., Lazarus P. (2014). Regulation of UDP-Glucuronosyltransferase 1A1 Expression and Activity by MiRNA 491–3p. J. Pharmacol. Exp. Ther..

[55] Papageorgiou I., Court M.H. (2017). Identification and Validation of MiRNAs Directly Regulating the UDP-Glucuronosyltransferase 1A Subfamily Enzymes by a Functional Genomics Approach. Biochem. Pharm..

[56] Li D., Knox B., Chen S., Wu L., Tolleson W.H., Liu Z., Yu D., Guo L., Tong W., Ning B. (2019). MiRNAs Hsa-MiR-495–3p and Hsa-MiR-486–5p Suppress Basal and Rifampicin-Induced Expression of Human Sulfotransferase 2A1 (SULT2A1) by Facilitating MRNA Degradation. Biochem. Pharm..

[57] Pan Y.-Z., Zhou A., Hu Z., Yu A.-M. (2013). Small Nucleolar RNA-Derived MiRNA Hsa-MiR-1291 Modulates Cellular Drug Disposition through Direct Targeting of ABC Transporter ABCC1. Drug Metab. Dispos..

[58] Pan Y.-Z., Morris M.E., Yu A.-M. (2009). MiRNA-328 Negatively Regulates the Expression of Breast Cancer Resistance Protein (BCRP/ABCG2) in Human Cancer Cells. Mol. Pharm..

[59] To K.K.W., Zhan Z., Litman T., Bates S.E. (2008). Regulation of ABCG2 Expression at the 3′ Untranslated Region of Its MRNA through Modulation of Transcript Stability and Protein Translation by a Putative MiRNA in the S1 Colon Cancer Cell Line. Mol. Cell Biol..

[60] El Saadany T., van Rosmalen B., Gai Z., Hiller C., Verheij J., Stieger B., van Gulik T., Visentin M., Kullak-Ublick G.A. (2019). MiRNA-206 Modulates the Hepatic Expression of the Organic Anion-transporting Polypeptide 1B1. Liver Int..

[61] Li X., Tian Y., Tu M.-J., Ho P.Y., Batra N., Yu A.-M. (2019). Bioengineered MiR-27b-3p and MiR-328–3p Modulate Drug Metabolism and Disposition via the Regulation of Target ADME Gene Expression. Acta Pharm. Sin. B.

[62] Li X., Pan Y.-Z., Seigel G.M., Hu Z.-H., Huang M., Yu A.-M. (2011). Breast Cancer Resistance Protein BCRP/ABCG2 Regulatory MiRNAs (Hsa-MiR-328, -519c and -520h) and Their Differential Expression in Stem-like ABCG2+ Cancer Cells. Biochem. Pharm..

[63] Wang Y., Tu M.-J., Yu A.-M. (2024). Efflux ABC Transporters in Drug Disposition and Their Posttranscriptional Gene Regulation by MiRNAs. Front. Pharm..

